# Reduction in the proportion of fevers associated with *Plasmodium falciparum *parasitaemia in Africa: a systematic review

**DOI:** 10.1186/1475-2875-9-240

**Published:** 2010-08-22

**Authors:** Valérie D'Acremont, Christian Lengeler, Blaise Genton

**Affiliations:** 1 Swiss Tropical and Public Health Institute, P.O. Box, 4002 Basel, Switzerland; 2 University of Basel, Basel, Switzerland; 3 Department of Ambulatory Care and Community Medicine, Infectious Disease service, University Hospital, Lausanne, Switzerland

## Abstract

**Background:**

Malaria is almost invariably ranked as the leading cause of morbidity and mortality in Africa. There is growing evidence of a decline in malaria transmission, morbidity and mortality over the last decades, especially so in East Africa. However, there is still doubt whether this decline is reflected in a reduction of the proportion of malaria among fevers. The objective of this systematic review was to estimate the change in the Proportion of Fevers associated with *Plasmodium falciparum *parasitaemia (PFPf) over the past 20 years in sub-Saharan Africa.

**Methods:**

*Search strategy*. In December 2009, publications from the National Library of Medicine database were searched using the combination of 16 MeSH terms.

*Selection criteria*. Inclusion criteria: studies 1) conducted in sub-Saharan Africa, 2) patients presenting with a syndrome of 'presumptive malaria', 3) numerators (number of parasitologically confirmed cases) and denominators (total number of presumptive malaria cases) available, 4) good quality microscopy.

*Data collection and analysis*. The following variables were extracted: parasite presence/absence, total number of patients, age group, year, season, country and setting, clinical inclusion criteria. To assess the dynamic of PFPf over time, the median PFPf was compared between studies published in the years ≤2000 and > 2000.

**Results:**

39 studies conducted between 1986 and 2007 in 16 different African countries were included in the final analysis. When comparing data up to year 2000 (24 studies) with those afterwards (15 studies), there was a clear reduction in the median PFPf from 44% (IQR 31-58%; range 7-81%) to 22% (IQR 13-33%; range 2-77%). This dramatic decline is likely to reflect a true change since stratified analyses including explanatory variables were performed and median PFPfs were always lower after 2000 compared to before.

**Conclusions:**

There was a considerable reduction of the proportion of malaria among fevers over time in Africa. This decline provides evidence for the policy change from presumptive anti-malarial treatment of all children with fever to laboratory diagnosis and treatment upon result. This should insure appropriate care of non-malaria fevers and rationale use of anti-malarials.

## Background

Currently, the global target for malaria control is to provide prompt and effective treatment as well as insecticide-treated nets (ITNs) to 80% of the people at risk of malaria by the end of 2010 [1,2]. The greatly increased malaria control effort since 2000 has been supported by global initiatives such as the Global Fund to fight AIDS, Tuberculosis and Malaria, the World Bank Malaria Booster Programme and the US Presidential Malaria Initiative. By the end of 2008, more than 50 African states had adopted artemisinin-combination therapy (ACT) and the number of ITNs distributed had increased more than 10 times in 14 African states [3]. There is evidence now of reduced malaria transmission, morbidity and mortality in locations where these strategies have been massively deployed [4,5]. There is also a documented decline in Africa of *Plasmodium falciparum *prevalence rates in children aged 2-10 years from 37% before the year 2000, to 17% after 2000 [6]. This decline is further evidenced in recent Demographic and Health Surveys (DHS) in malaria endemic countries of sub-Saharan Africa: 11 of the 12 national surveys conducted since 2004 showed declines in underfive mortality estimates over the previous five years (declines of 5% to 30%, median 23%) [7].

Theoretically, a reduction of malaria transmission, and hence parasitaemia, should translate into a decline of the proportion of fevers due to malaria, but the relationship between these two parameters is not straightforward. In part, this is due to the fact that the pattern of other causes of fever found in each area or patient population is not uniform and influence, therefore, the magnitude of the effect. Annual episodes of fever among African children have been estimated to be as high as 870 million [8]. For those patients who reach clinics across the continent, a presumptive diagnosis of malaria is done in 30-40% of the cases [9]. Malaria thus appears to be the number one cause of fever in sub-Saharan Africa, as well as the leading cause of mortality, at least in children [10].

However, these data might be largely overestimated nowadays due to the lack of specificity of a purely clinical diagnosis. Assigning malaria as a cause of fever in the absence of laboratory diagnosis is based on clinical experience but also on an understanding of the underlying epidemiology of the disease. Unfortunately, current practice by health care providers largely ignores the declining trend of *P. falciparum *parasite prevalence observed in community surveys and the proportion of fevers attributed to malaria does not seem to change. This question is of great practical relevance to correctly estimate the burden of disease due to malaria and for tracking progress in malaria control.

The objective of the present project was, therefore, to review the available evidence on the proportion of fevers due to malaria over the last 20 years in sub-Saharan Africa, to identify trends and to quantify the magnitude of expected changes. This assessment has obvious implications for optimizing recommendations concerning the management of fever cases in children under five years of age who live in highly endemic areas.

## Methods

### Criteria for considering studies for this review

#### Type of studies: observational studies or diagnostic studies

Inclusion criteria were i) study conducted in an area of sub-Saharan Africa where *P. falciparum *is the dominant species, ii) including patients (or a clear subset of patients) presenting at a health facility with a syndrome of 'presumptive malaria', either considered as such by the health worker in charge, or defined on clinical criteria by the investigators, iii) numerator (number of parasitologically-confirmed cases) and denominator (total number of presumptive malaria cases) available or possible to calculate from text, tables, or obtained after request to the authors, iv) good quality microscopy (reference centre or research laboratory), v) no obvious selection bias of patients.

Exclusion criteria were: i) studies using a parasite density threshold for malaria case definition, ii) intervention studies and studies aimed at evaluating the incidence of malaria episodes, iii) studies including < 100 patients, or those focusing on inpatients only and severe malaria. The reasons for excluding these studies are as follows: i) defining a parasite threshold leads to a patients population that has lower proportion of malaria than when presence/absence of parasitaemia is used to define a case, ii) messages given to subjects in interventions studies ('attend as soon as possible in case of any symptom') lead to a patients population that is likely to be biased towards milder cases, and hence potentially lower prevalence of parasitaemia (undetectable parasitaemia because of low density), iii) studies with < 100 patients would have lacked precision (more than +/- 10% in the prevalence estimate) and be at higher risk of bias and confounding because of the limited sampling.

#### Type of participants

Patients of any age

#### Type of outcome measures

Proportion of fevers associated with *Plasmodium falciparum *parasitaemia (PFPf).

### Search method for identification of studies

All relevant published studies of human medicine, regardless of language were searched for. In the COCHRANE reviews, 'malaria' as search term was used. In MEDLINE, a combination of several MeSH terms was used in the following way: malaria AND Africa AND (diagnosis OR parasitemia OR microscopy) AND (epidemiology OR sensitivity and specificity OR prevalence OR seasons OR transmission OR cross-sectional studies OR predictive value of tests) AND (aetiology OR fever OR algorithms OR case management). Titles and abstracts to be reviewed were listed. On the basis of abstract reading, full papers were selected, reviewed and those that matched the selection criteria were retained. Then, abstracts of the related articles of this first series of papers were explored, reviewed and eventually the full paper was read if deemed appropriate. All references of the retained papers were also examined. This process was performed iteratively until no new suitable study could be found.

#### Databases

Cochrane Infectious Diseases Group Specialized Register (up to 15^th ^December 2009), MEDLINE (through 15^th ^December 2009).

#### Researchers and organizations

The following authors were contacted for clarifications on entry criteria of patients or for additional information on proportion of malaria among their sample: Pedro Alonso, Patrick Kachur, Christoph Hatz, Sophie Yacoub, Babacar Faye, Thomas Mschana and Nadjitolnan Othingué.

#### Reference lists

The reference lists of all studies identified by the above methods was checked.

### Methods of the review

#### Study selection

One author (VdA) independently applied the inclusion criteria to all identified studies. All studies selected were checked by a second author (BG) for appropriateness. For studies for which there were doubts about inclusion, the second author assessed them fully and potential differences were discussed until consensus was reached.

#### Data extraction

Besides the proportion of fevers with associated *Plasmodium falciparum*, eight variables were extracted from each paper: year, season (rainy versus dry; when season was not mentioned it was searched on the CIA website [11] using dates of beginning and end of recruitment), country, setting (urban versus rural), health facility type (hospital versus primary care), age group (< 5 years, 5-15 years, adults), clinical inclusion criteria used and total number of patients.

#### Data analysis

As it was not possible to obtain the original databases of all studies, essentially a descriptive analysis on aggregated data was performed. The proportion of fever or presumptive malaria cases associated with *P. falciparum *documented by high quality microscopy, hereinafter referred to as proportion of fevers due to malaria (PFPf), was retrieved or calculated from each study. To assess the trend of PFPf over time, the median PFPf including all studies (pooled analysis) was compared for the period up to the year 2000, and for that from 2001 onwards. This threshold was chosen since large-scale interventions started around this time thanks to a massive increase in funding for control measures [12]. That cut-off is also consistent with the analysis by Guerra *et al *[6] for parasitaemia. No formal multivariate analysis was possible since individual records for each study were not available. However, to investigate potential confounding factors, a stratified analysis by age group (< 5 and ≥5 years), season (rainy and dry), setting (rural and urban), type of health facility (primary care and hospital) was performed using data from studies where such information was available. These parameters are known to have an effect on malaria fevers and the categories are standard to describe the epidemiology of malaria.

## Results

### Description of studies

**Figure 1:**
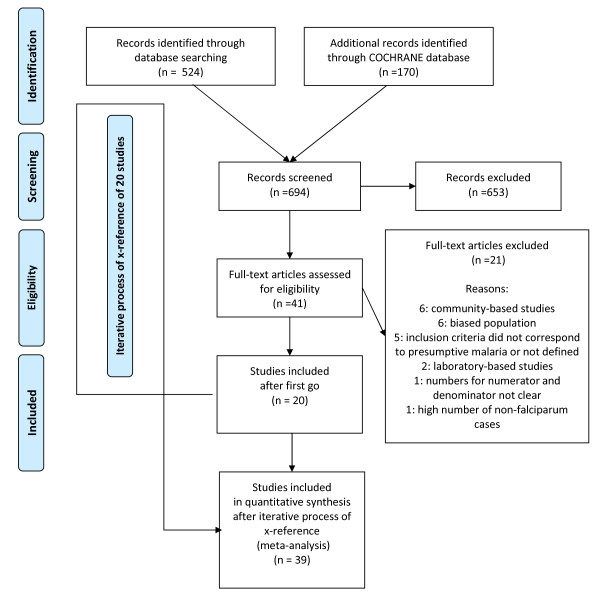
**PRISMA flow chart for literature search**.

Up to 15^th ^December 2009, 170 titles were extracted from the COCHRANE database, but none was relevant for this review. From MEDLINE, 524 titles were identified and extracted, all abstracts were reviewed and 41 papers were selected in the first round. Based on the reading of the full article, 20 met all inclusion criteria and were retained. After iterative cross-referencing of these 20 articles, 19 additional articles meeting all inclusion criteria were found (see Figure 1). The 39 studies included in the final analysis were conducted between 1986 and 2007 and published between 1989 and 2009. The total number of patients included was 42,979 (median: 576; range: 149 to 7713). All relevant details of the included studies are described in Table 1.

**Table 1:** All 39 included studies showing the Proportions of Fevers associated with *Plasmodium falciparum *parasitemia (PFPf)

First author	Start of the study	Country	Urban/rural area	Place of recruit-ment	Clinical inclusion criteria	Total number of patients	Overall PFPf	Age groups	*Season*	PFPf
Rooth [13]	1986	Tanzania	Rural	Primary care	History of fever	596	81%	≤ 9 years	Both seasons	81%

Rougemont [22]	1987	Niger	Rural	Primary care	Temp ≥37.5°C	285	57%	≤ 9 years	Rainy season	57%

Salako [23]	1987	Nigeria	Urban	Hospital	History of fever or elevated temp	7713	55%	< 5 years	Both seasons	61%
										
								5-18 years		60%
										
								> 18 years		41%

Ejezie [24]	1988	Nigeria	Urban	Hospital	Presumptive malaria	1188	45%	≤ 9 years	Both seasons	45%

Gaye [25]	1988	Senegal	Urban	Primary care	Temp ≥38°C	353	31%	≤ 9 years	Rainy season	28%
										
								10-14 years		35%
										
								> 14 years		33%

Olivar [26]	1989	Niger	Urban	Primary care	Temp > 38°C	576	35%	< 5 years	Rainy season	62%
									
									Dry season	5%

Lubanga [27]	1992	Uganda	Urban	Hospital	History of fever	435	64%	≤ 5 years	Rainy season	64%

Meremikwu [28]	1993	Nigeria	Urban	Hospital	Temp ≥37.5°C	225	63%	< 5 years	Rainy season	63%

Redd [29]	1993	Malawi	Rural	Hospital	Presumptive malaria	1124	60%	< 5 years	Rainy season	60%

Olaleye [30]	1993	the Gambia	Rural	Primary care	History of fever or temp ≥37.5°C	407	59%	≤ 9 years	Rainy season	59%

Weber [31]	1993	the Gambia	Urban	Hospital	Presumptive malaria	440	7%	< 5 years	Rainy season	17%
									
									Dry season	4%

Gaye [32]	1994	Senegal	Urban	Hospital	History of fever	762	31%	All ages	Rainy season	59%
									
									Dry season	5%
								
								< 5 years	Both seasons	14%
										
								≥ 5 years		36%

Font [14]	1995	Tanzania	Rural	Primary care	History of fever or temp > 37.5°C	641	44%	< 5 years	Rainy season	57%
										
								≥ 5 years		24%

Tarimo [33]	1996	Tanzania	Urban	Hospital	Presumptive malaria	400	52%	All ages	Both seasons	52%

Cooke [34]	1996	the Gambia	Rural	Hospital	History of fever or elevated temp	398	36%	All ages	Rainy season	36%

Muhe [35]	1996	Ethiopia	Rural	Primary care	History of fever or temp ≥38°C	2490	22%	< 5 years	Rainy season	30%
									
									Dry season	6%

Cortes [36]	1996	Mauritania	Urban	Hospital	History of fever	416	19%	All ages	Both seasons	19%

Oster [37]	1996	Tanzania	Rural	Hospital	History of fever or temp ≥37.5°C	168	14%	Adults	Rainy season	20%
									
									Dry season	0%

Nsimba [15]	1997	Tanzania	Rural	Primary care	Presumptive malaria	449	38%	≤ 5 years	Rainy season	38%

Akim [38]	1997	Tanzania	Rural	Primary care	History of fever	6580	43%	≤ 6 years	Both seasons	57%
										
								7-15 years		44%
										
								> 15 years		27%

Arness [39]	1998	Kenya	Rural	Primary care	Presumptive malaria	2796	29%	< 5 years	Both seasons	32%
										
								5-15 years		35%
										
								> 15 years		23%

Tarimo [40]	2000	Tanzania	Rural	Hospital	Malaria based on IMCI criteria	395	70%	≤ 5 years	Rainy season	70%

Guthmann [41]	2000	Uganda	Rural	Hospital	Presumptive malaria	742	57%	< 5 years	Rainy season	64%
										
								> 5 years		52%

Gouagna [42]	2000	Kenya	Rural	Primary care	Presumptive malaria	3754	47%	< 5 years	Both seasons	55%
										
								5-15 years		57%
										
								> 15 years		36%

Raharimalala [43]	2001	Madagas-car	Rural	Primary care	Presumptive malaria	149	35%	< 15 years	Rainy season	37%
										
								≥ 15 years		29%

Assoumou [44]	2001	Ivory Coast	Urban	Hospital	Temp ≥37.5°C	902	29%	< 5 years	Both seasons	31%
										
								5-15 years		27%

Othnigue [45]	2002	Chad	Urban	Primary care	Presumptive malaria	712	30%	All ages	Rainy season	35%
									
									Dry season	12%
								
								< 5 years	Both seasons	19%
										
								5-14 years		50%
										
								≥ 15 years		27%

Zurovac [46]	2002	Kenya	Rural	Primary care	Sent for malaria test by clinician in charge	261	13%	≥ 5 years	Rainy season	13%

Malik [47]	2002	Sudan	Urban	Hospital	History of fever	655	12%	< 5 years	Dry season	11%
										
								5-16 years		13%

Wang [48]	2002	Ivory coast	Urban	Primary care	History of fever or temp ≥37.5°C	429	35%	≤ 5 years	Rainy season	36%
										
								6-15 years		44%
										
								> 15 years		26%

Wang [49]	2002	Burkina	Urban	Primary care	History of fever or temp ≥37.5°C	560	22%	≤ 5 years	Dry season	22%
										
								6-15 years		37%
										
								> 15 years		18%

Wang [50]	2003	Tanzania	Urban	Primary care	History of fever or temp ≥37.5°C	717	5%	≤ 5 years	Dry season	5%
										
								6-15 years		7%
										
								> 15 years		4%

Wang [51]	2003	Benin	Urban	Primary care	History of fever or temp ≥37.5°C	379	2%	≤ 5 years	Dry season	4%
										
								6-15 years		0%
										
								> 15 years		1%

Yacoub [52]	2003	Zanzibar	Rural	Primary care	History of fever or temp > 37.5°C	207	77%	≤ 5 years	Rainy season	77%

Ogungbami-gbe [53]	2004	Nigeria	Urban	Hospital	Temp ≥37.5°C	646	53%	≤ 9 years	Rainy season	62%
									
									Dry season	28%

Kachur [54]	2004	Tanzania	Rural	Primary care	History of fever	769	31%	< 5 years	Rainy season	43%
										
								≥ 5 years		23%

Reyburn [16]	2005	Tanzania	Rural	Hospital	Sent for malaria test by clinician in charge	2397	15%	< 5 years	Rainy season	21%
										
								5-15 years		17%
										
								> 15 years		8%

Reyburn [55]	2005	Tanzania	Rural	Hospital	Sent for malaria test by clinician in charge	214	4%	All ages	Rainy season	4%

Mens [56]	2007	Kenya	Rural	Primary care	History of fever or temp ≥37.5°C	650	17%	≤ 12 years	Rainy season	17%

#### Time frame

24 studies were conducted up to the year 2000 and 15 from 2001 onwards.

#### Location

**Figure 2:**
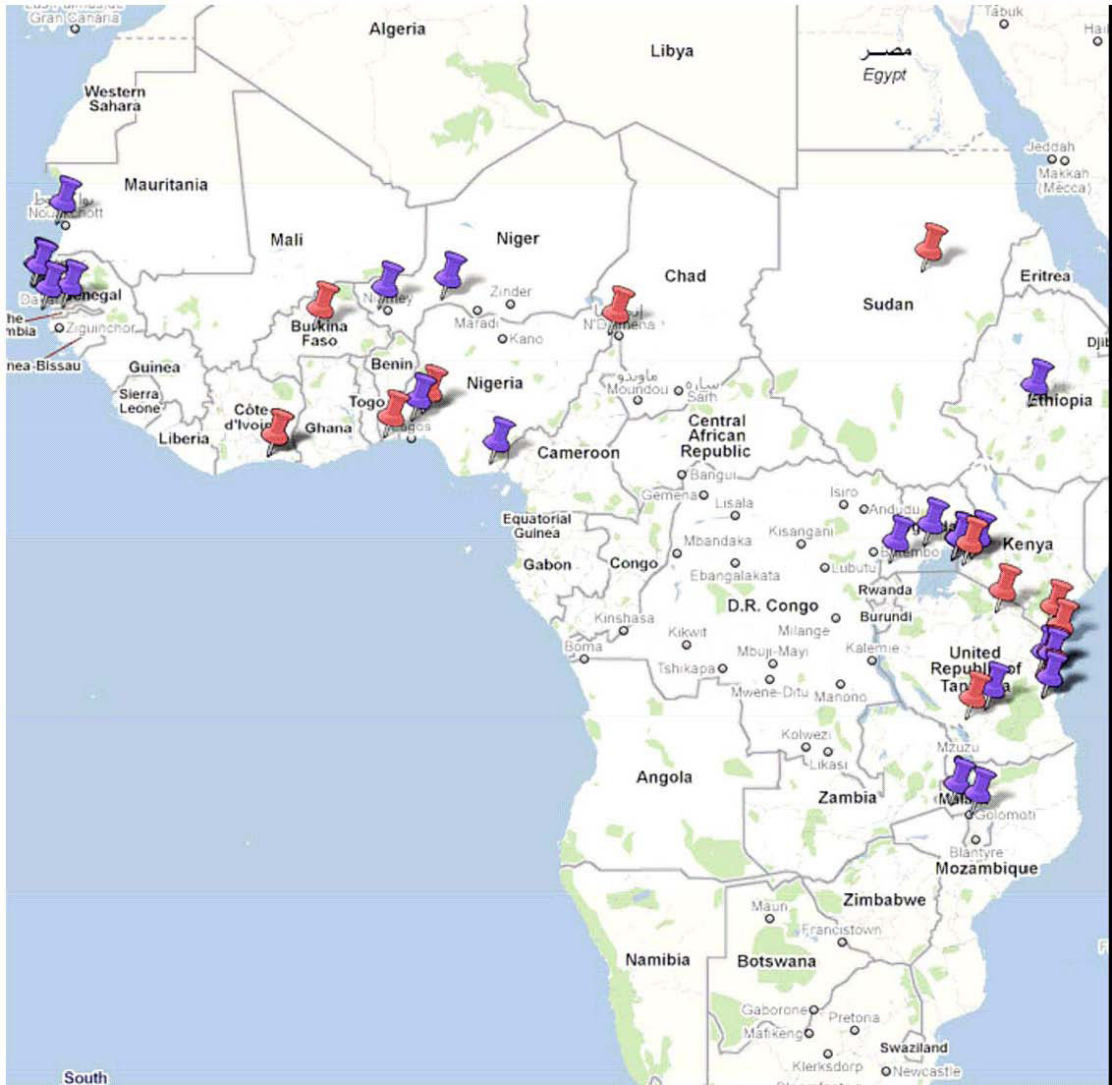
**Geographical distribution of sites of included studies (blue pins are for studies ≤ year 2000 and red pins > year 2000)**.

The included studies were conducted in 16 different African countries. 21 studies took place in East Africa, mainly Tanzania (12 studies), 16 studies in West Africa, one in Central Africa (Chad) and one in the Northern region (Sudan) (Figure 2).

#### Age group

Fifteen studies included children only (age < 5 years, n = 8; age < 9 years, n = 6; age < 12 years, n = 1). One study included only adults older than 18 years and one study only patients older than 5 years. The remaining 22 studies included patients of all ages.

#### Level of endemicity and transmission season

Studies on clinical management of malaria have been done mainly in highly endemic areas and a period of the year with peak transmission, and this is reflected in the included studies. Patients were recruited during the rainy season only, the rainy and the dry seasons and the dry season only in 19, 16 and 4 studies, respectively.

#### Setting and level of health care

Twenty one studies were conducted in a rural area of Africa (54%), while the remaining took place in urban areas. Also, 21 studies were undertaken at primary care level and the rest at hospital level.

### Overall proportion of parasitaemia among fever cases (PFPf)

The proportion of parasitaemia among fever or presumptive malaria cases (PFPf) varied considerably between studies and sub-groups of patients within the same study. Taking each study as a unit, the median PFPf was 35% [inter-quartile range - IQR: 20-54%; range 2-81%).

### Variation of PFPf according to key stratification parameters

#### Age group-specific PFPf

Taking into account all studies where data were stratified by age or those that included only one defined age group, overall median PFPf was 36% (IQR 21-60%; range 4-77%) for children under five years (25 studies) and 26% (IQR 13-33%; range 1-53%) for those above five years (18 studies). In the 16 studies providing stratified values of PFPf in both age groups, median PFPf in children under five years of age (32%; IQR 18-55%) was not significantly different from that in the group above five years (27%; IQR 19-34%). When using only the 10 studies providing details for the older age groups, the median PFPf was 27% (IQR 20-50%) for the group under 5 years, 40% (IQR 22-48%) for the age group of 5-15 years, and 24% (IQR 11-27%) in adults above 15 years.

#### Season-specific PFPf

The overall median PFPf was 37% (IQR 30-60%; range 4-77%) in the rainy season versus 5% (IQR 4-12%; range 0-28%) in the dry season. Among all factors studied, this was the most dramatic difference observed. This difference remained when restricting the analysis to the 7 studies providing stratified value of PFPf for both the rainy and dry seasons: 35% (IQR 25-57%) versus 5% (IQR 4-9%).

#### Setting-specific PFPf

##### Urban/rural

There were 21 studies conducted in rural areas and 18 in urban areas (as defined by investigators). The overall median PFPf was 38% (IQR 22-57%; range 4-81%) in rural areas versus 31% (IQR 19-50%; range 2-64%) in urban settings.

##### Level of health care

21 studies were conducted in primary care facilities and 18 in the outpatient department of a hospital. The overall median PFPf was 35% (IQR 22-44%; range 2-81%) in primary care settings versus 40% (IQR 16-56%; range 4-70%) in hospitals.

### PFPf up to the year 2000 and afterwards

**Figure 3:**
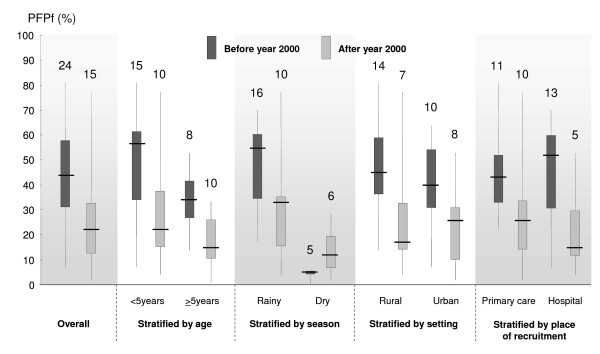
**Comparison between the Proportions of Fevers associated with *Plasmodium falciparum *parasitemia (PFPf) in years ≤2000 and > 2000, stratified by baseline characteristics (numbers above plots corresponds to the number of studies involved)**.

When comparing PFPf from studies conducted up to the year 2000 (24 studies) with those done afterwards (15 studies), there was a clear reduction in the median PFPf from 44% (IQR 31-58%; range 7-81%) to 22% (IQR 13-33%; range 2-77%) (Figure 3). This dramatic decline is likely to reflect a true change since all variables listed above and which were shown to have an effect on PFPf were well balanced between the two groups of studies. For studies before and after 2000 respectively, 17.5/24 (73%) and 9.5/15 (63%) studies were conducted during the rainy season (when a study took place during both the rainy and the dry season, the count was 0.5 study for each of the season) (p = 0.45); 14/24 (58%) and 7/15 (47%) studies were conducted in a rural setting (p = 0.53); and 11/24 (46%) and 10/15 (67%) were conducted at primary care level (p = 0.32).

In the 11 studies from Tanzania (considered mostly as a highly endemic area), PFPf in children under five years during the rainy season decreased from 81% in 1986-88 [13] to 57% in 1995 [14], 38% in 1997 [15] and 21% in 2005 [16].

To further check for potential confounding factors, a stratified analysis was conducted including each of the variables listed above. The reduction of PFPf over time was confirmed since median PFPfs were almost always lower for the years after 2000 compared to the years ≤2000 (see Figure 3). There was one exception in the case of the data collected during the dry season.

## Discussion

This systematic review demonstrates a 50% reduction of PFPf for the period after year 2000 when compared to that of ≤2000 (22% versus 44%). This decrease by half of the proportion of malaria cases among fever episodes is likely to be due to a reduction of malaria transmission. It mirrors the reduction observed in parasite prevalence rates collected from community cross-sectional surveys in sub-Saharan Africa during the same time periods (17% after year 2000 versus 37% before) [6]. A decrease in malaria is now observed in many settings across sub-Saharan Africa, mainly because of the large scale implementation of effective control measures following a drastic increase of funding since the year 2000 [12]. Recently, Okiro & Snow demonstrated the clear linear relationship between the risk of infection among febrile children and parasite prevalence in the community [17].

An inherent difficulty with systematic reviews is that they look at studies which may not be comparable. In the present review, the most recent studies report a lower proportion of malaria attributable fevers, but some of these later studies have been conducted in areas of lower endemicity than those performed previously. This is especially true for some studies in Tanzania, a country largely represented in this systematic review. In part, this was due to the fact that some of the recent studies were conducted in urban and peri-urban settings, whereas old ones were traditionally done in rural places, where transmission of malaria is usually higher. Part of the reduction in the proportion of malaria among fevers may thus be ascribed to selection biases. To address this problem, stratified analyses for variables known to have an effect on PFPf were performed. These analyses confirmed the reduction of PFPf over time when controlling for these different factors. The only exception was for dry season and this is easily explainable by the fact that the median PFPf was always rather low, irrespective of the period. When data are available, such as in Tanzania for example, the substantial reduction of PFPf parallels closely the decline of incidence of malaria episodes and overall mortality in children under five observed in the same areas over the last fifteen years [18,19] (Khatib *et al*, unpublished data).

Although the reduction of PFPf was considerable, it is unclear how representative these data are at this point in time. Data are over-represented in sites with research institutions or special situations and it would be a great importance to confirm such trends in other settings, especially in the largest malarious countries on the continent, the Democratic Republic of the Congo and Nigeria. Also, within a country or region, the reductions are not necessarily uniform. There are still a number of places that harbour PFPf over 50%, especially so in areas where healthcare is not readily accessible.

On the other hand, there is no doubt that malaria control is very successful in many countries [5] and these trends are expected as a result of greatly improved preventive activities. In any case, the immediate practical implication of the changing epidemiology of fever episodes is the much increased need for a systematic laboratory diagnosis of every case before initiating treatment for malaria [20].

## Conclusions

This systematic review demonstrates a considerable reduction of PFPf in sub-Saharan Africa between the periods before the year 2000 and from 2001 onwards (44% versus 22%). With only around a fifth of all fever episodes being associated with malaria parasitaemia, this review provides strong evidence to support the new WHO policy of laboratory-based diagnosis and treatment upon result [21]. This should insure appropriate care of non-malaria fevers and rationale use of anti-malarials. The relative cost effectiveness and value of introducing diagnosis for febrile children will obviously depend on infection prevalence in the community.

## Competing interests

The authors declare that they have no competing interests.

## Authors' contributions

VdA did the literature search, the analyses, and wrote the first draft of the manuscript. BG counterchecked the selection of articles and contributed to the manuscript writing. CL contributed to the manuscript. All authors read and approved the final manuscript.

## Financial disclosure

VdA was supported by a grant of the Swiss National Science Foundation (Grant # 3270B0-109696). CL and BG have permanent positions in their own institution. The funder had no role in study design, data collection and analysis, decision to publish, or preparation of the manuscript.
